# Draft Genome Sequence of the Bacteriophage vB_Eco_slurp01

**DOI:** 10.1128/genomeA.01111-16

**Published:** 2016-11-17

**Authors:** Pavelas Sazinas, Carrie Smith, Aqilah Suhaimi, Jon L. Hobman, Christine E. R. Dodd, Andrew D. Millard

**Affiliations:** a Microbiology and Infection Unit, Warwick Medical School, University of Warwick, Coventry, United Kingdom; b School of Life Sciences, University of Warwick, Coventry, United Kingdom; c School of Biosciences, University of Nottingham, Sutton Bonington Campus, Sutton Bonington, United Kingdom

## Abstract

Bacteriophage vB_Eco_slurp01 was isolated from porcine feces using *Escherichia coli* MG1655 as a host. With a genome size of 348 kb, vB_Eco_slurp01 is one of the largest bacteriophages isolated to date.

## GENOME ANNOUNCEMENT

There are approximately 4.4 million pigs in the United Kingdom, with an estimated £212 million export value (1). Yet there are relatively few studies on the virome of pigs. This study aimed to begin investigating the diversity of bacteriophages in porcine feces. Here, we isolated and sequenced the bacteriophage vB_Eco_slurp01, which is capable of infecting *Escherichia coli* MG1655.

Bacteriophage genomic DNA was prepared from cultures using a phenol:chloroform extraction method (2). One nanogram of genomic DNA was prepared using the Nextera XT DNA sample preparation kit (Illumina) prior to sequencing on the MiSeq platform using V2 (2 × 250-bp) chemistry. The resulting FASTQ files were assembled with SPAdes version 3-7 with the “-careful” option (3). The genome was sequenced to an average depth of 381×. The resultant single contig was annotated with Prokka version 1.11 using a custom database constructed from all complete viral genomes within the European Nucleotide Archive (4). ROARY was used to identify core genes between the phages vB_Eco_slurp01, PBECO4, and 121Q (5). Bacteriophage vB_Eco_slurp01 had a genome size of 348 kb with 588 coding sequences, seven tRNAs, and a GC content of 34.05%. There are currently only eight bacteriophage genomes that are larger than 300 kb in size; these infect a range of bacteria, including *Bacillus*, *Aureococcus*, *Cronobacter*, *Enterobacter*, *Escherichia*, and *Pseudomonas* spp. Thus, vB_Eco_slurp01 adds to this small number of “jumbo” phages. At the nucleotide level, vB_Eco_slurp01 is similar to the coliphages PBECO4 (KC295538.1) (6) and 121Q (KM507819.1) with an average nucleotide identity of 98.4% and 96.46%, respectively, across the genome. This high level of identity is surprising given that these phages were isolated from geographically distant regions: Gwacheon, South Korea (PBECO4), Quebec, Canada (121Q), and Nottinghamshire, United Kingdom (vB_Eco_slurp01).

Furthermore, these phages all share a high degree of synteny across their genomes. Comparison of gene content between the three strains revealed a core gene set of 405 genes. While conserved between isolates, the majority of these genes encode hypothetical proteins. As with previous jumbo phages, a number of bacterial host homologue genes were detected, including genes coding for a σ^54^ modulation protein, RpoD, GyrA, GyrB, and ribonucleoside-diphosphate reductase subunits. Although homologous to *E. coli* genes, many of the coding sequences had greater similarity to genes from other bacteria; for example, *gyrB* had higher similarity to *Bacteriovorax* sp. DB6_IX than *E. coli*. In addition, a gene encoding a putative tellurite resistance protein (TelA) was also observed. This is a feature that is also common to the phages PBECO4 and 121Q. Intriguingly, the resistance protein is associated with Gram-positive bacteria (7) and is not part of the tellurite resistance operon found in *E. coli* (8).

The genome of vB_Eco_slurp01 provides further insights into the small number of bacteriophages that have genomes greater than 300 kb in size. Furthermore, this study demonstrates that double-stranded DNA bacteriophages from distant geographical regions have highly conserved genomes.

### Accession number(s).

The draft genome sequence of bacteriophage vB_Eco_slurp01 has been deposited in DDBJ/ENA/GenBank under the accession number LT603033.
